# Placebo Studies and Patient Care: Where Are the Nurses?

**DOI:** 10.3389/fpsyt.2021.591913

**Published:** 2021-03-15

**Authors:** Marco Annoni, Sarah Buergler, Sif Stewart-Ferrer, Charlotte Blease

**Affiliations:** ^1^Centro Interdipartimentale per l'Etica e l'Integrità nella Ricerca, National Research Council of Italy, Roma, Italy; ^2^Fondazione Umberto Veronesi, Milano, Italy; ^3^Division of Clinical Psychology and Psychotherapy, Faculty of Psychology, University of Basel, Basel, Switzerland; ^4^Research Unit of General Practice, Department of Public Health, Faculty of Health Sciences, University of Southern Denmark, Odense, Denmark; ^5^Division of General Medicine and Primary Care, Beth Israel Deaconess Medical Center and Harvard Medical School, Boston, MA, United States

**Keywords:** placebo and nocebo effects, prescription, nursing, clinician-patient relationship, medical ethics, primary care, general practice, contextual factors

## Introduction

Placebo research has gained traction in recent years as empirical researchers have begun to shed light on the psychobiological mechanisms underpinning placebo and nocebo effects [e.g., (1, 2)]. A considerable body of evidence now demonstrates that placebo and nocebo effects are ubiquitous in research and clinical settings and may significantly modulate patients' symptoms in prevalent conditions such as pain, migraine, depression, or irritable bowel syndrome (3–5). Following these empirical breakthroughs, experts have begun to advocate new therapeutic protocols aimed at improving patients' care through attention to placebo and nocebo effect in clinical settings (6).

So far, however, placebo studies in medicine are largely doctor-centric. While a growing body of research has focused on physicians (and also clinical psychologists and psychotherapists) the literacy, understanding, and attitudes about placebo effects and placebo usage among medical professionals such as nurses, nurse practitioners, and physician assistants have been comparatively less explored. This gap may be due to different factors, such as the incorrect assumption in some regions that, unlike doctors, nurses do not prescribe placebos and do not need to know about placebo/nocebo effects. Yet this view is problematic as non-physician professionals are no less likely to confront clinical situations in which they may influence patient's outcomes through placebo and nocebo effect modulation, and the use of placebos. For example, worldwide many nurses are currently involved in the mass-administration of Covid-19 vaccines, with the possible scope to influence placebo effects among recipients. Furthermore, in their clinical work, non-physician professionals may face ethical and practical challenges related to these effects that significantly differ from those confronted by doctors. In this *Opinion* our aim is to motivate further empirical research, and ethical debate, specifically with respect to the role of nursing health professionals in influencing placebo and nocebo effects.

## Background: Placebo Studies and Primary Care

A *placebo* is any intervention (drug, pill, injection, etc.) that, despite being considered a sham, is nonetheless administered as if it were effective. Placebos fulfill different roles in medicine (7). In research, placebos are typically used as controls in clinical trials. In clinical settings, they may serve a variety of other purposes, from placating nervous patients to providing symptomatic relief via “placebo effects” (8). Placebos can be described as “pure” if they have no pharmacological effects (e.g., saline injections), or as “impure” if they have pharmacological effects but not for the illness or symptoms being treated (e.g., antibiotics for a viral infection).

*Placebo effects* are real and beneficial effects (e.g., a decrease of pain) that may occur in response to salient features of the healing context—such as the provision of a treatment (sham or real) or other aspects of the therapeutic relationship, e.g., the doctor-patient interaction (through verbal and non-verbal communication) (9). In contrast, *nocebo effects* are understood to modulate patients' symptoms in negative ways (10). Placebo and nocebo effects have been shown to occur through various psychobiological mechanisms—for example, classical conditioning and expectancies (11).

To date, a wide range of quantitative and qualitative surveys have been conducted among physicians in the US, UK, Europe, and Australia (12–14). These surveys have predominantly explored the level of understanding among primary care physicians (PCPs) or general practitioners (GPs) about placebo concepts, including their practices, knowledge, and attitudes in relation to placebo use, and placebo and nocebo effects. Research suggests that placebo use by physicians is common in primary care; for example, in 2013, a large-scale UK survey found that 77% of GPs prescribed impure placebos at least once per week (13). In 2019, a survey in Australia found that 39% of surveyed GPs had prescribed pure placebos, and 77% had prescribed impure placebos (15). In 2020, in a US survey some PCPs admitted prescribing placebos more often for pain disorders, functional disorders, or for medically unexplained symptoms than for other medical conditions (16).

Intentionally prescribing placebos to elicit placebo effects can involve deception or opaque disclosures that may infringe on patients' right to informed consent, autonomy, and compromise trust (17, 18). For this reason, current ethical guidelines tend to discourage or forbid the administration of placebos in clinical contexts. The American Society for Pain Management Nursing, for instance, urges not to use placebos outside of clinical trials (19), while the American Medical Association considers the clinical use of placebos unethical without patient's “prior consent” (20). Recent studies have demonstrated that placebo effects may occur without placebos (21), deception (22) or when only possessed, but not applied (23). However, today there exists a gap between clinicians' views, experiences and attitudes about placebo concepts, and expert placebo researchers' consensus about best clinical practices (6).

## Rationale for Studying Nurses' Views

Nurses represent a major component of the worldwide medical force. Globally nurses outnumber doctors by a 2:1 ratio (9.2 million doctors vs. 18.1 million nurses)—although this ratio varies between countries (24). Depending on their licensure, in some countries nurse practitioners already substitute for doctors when it comes to prescribing medications (25). Also, there is a growing body of literature related to nurse prescribing (26).

Moreover, the role and proportion of nursing staff is expected to increase in light of systemic shifts in health care due to increasing costs, a shortage of primary care physicians, and an aging population [e.g., (27, 28)] Indeed, in many key areas of primary care such as hospices and geriatric care, nurses represent the first line of intervention when patients report pain or other non-specific symptoms. In a qualitative study by Courtenay et al. (29), patients valued nurses' approachability and interest in their well-being. A review evaluating the impact of nurses working as doctors' substitutes, indicates that nurse-led primary care leads to equal, or even better quality of care and higher patient satisfaction compared with doctor-led care, and that consultation length and number of attended return visits tend to be higher for nurses than for doctors (30). All these factors (i.e., time spent with patients; cues of practitioner warmth and empathy) have been associated with enhanced placebo effects (31). On the other hand, nocebo effects could be inhibited by selecting appropriate verbal disclosures during diagnosis and treatment (6). However, there is a great heterogeneity of nurses worldwide in terms of qualification, background, roles, and culture, which limit the findings of this review and should be considered in future studies.

Building on these findings, nurses may be especially well-placed to play an important role in augmenting placebo effects and inhibit nocebo effects in clinical interactions. Given their pivotal role in healthcare, we suggest that exploring the understanding, attitudes, and views of nurses (and other non-physician health professionals, such as physician assistants) is long overdue.

## What We Know

Research on nurses' views and experiences of placebos and placebo effects is remarkably under-represented in both placebo and nursing literature (32). In January 2021 we conducted a systematic literature search in PubMed using the terms “nursing,” “placebo effects,” and “education.” The search identified 178 results published in the last 10 years, of which only four papers were judged to be relevant. Of these, only one study (based in Saudi Arabia) compared the views of medical and nursing students on the placebo effect and found that nursing students had significantly higher knowledge of placebo effects and were more likely to believe in the effectiveness of placebos, compared with medical students (33).

Broadening the scope of our search, we found other authors acknowledge the role of nurses in the clinical context as prominently positioned to modulate placebo and nocebo effects [e.g., (32, 34, 35)]. Recent preliminary, cross-sectional surveys in Italy report that nurses are aware of the importance of contextual factors in patient care [i.e., verbal and non-verbal communication, psychical contact or empathetic therapeutic alliance; (36, 37)]. Similarly, surveys of Italian nursing students (38) and physical therapists (39) found that respondents already exploit contextual factors to modulate placebo effects and increase treatment outcomes in various conditions.

However, we are aware of no recent survey that has directly investigated the views of qualified nursing staff on placebo and nocebo effects. Existing surveys with a direct focus on the prescription of placebos (e.g., pills or saline injections) among nurse respondents are sparse. The majority of survey research was conducted over three decades ago, with studies restricted to questionnaire-based methods and small sample sizes. Exploring the rationale behind prescription, most surveys mention pain relief as a leading reason to administer placebos (40–46). Nurses also reported using placebos for diagnostic purposes—[e.g., to differentiate “organic” from “functional” disorders (40, 43, 44)]. Finally, and notably, Fässler et al. (47) identified in their systematic review that considerably more nurses used “pure” placebos during their professional life compared with physicians. The majority of nurses considered placebos ethically problematic, but few physicians and nurses thought that the use of placebos should be categorically prohibited (47).

## Addressing the Knowledge Gaps

There are three main areas where survey research might help to motivate, and inform, greater understanding of nurses' views about placebo use, and the modulation of placebo/nocebo effects, in nurse-patient interactions (see Table 1). First, more data is required to understand “placebo literacy” among nursing staff, in particular to explore nurses' understanding of the conceptual differentiation between clinical and research placebos, and their level of awareness with respect to scientific research into placebo and nocebo effects. Second, the experiences, and practices of nursing staff with regard to placebo use is largely missing from ethical discussion. Relatedly, we are aware of no studies that have probed nurses' perceptions or experiences of observing physicians' practices in relation to deceptive placebo use. Third, there is limited understanding of the attitudes of nurses with respect to the use of placebos and modulating placebo and nocebo effects in clinical care. For example, what are the attitudes with respect to practice around the use of deceptive placebos? Do nurses believe they have a professional obligation to maximize placebo effects, and mitigate nocebo effects? Researchers could avail of current methods of placebo/nocebo research (i.e., focus group interviews, questionnaires) to explore different nursing professions' attitudes and opinions. To initiate fuller appraisal of the implications of placebo and nocebo research for ethical practice, the social scientific research agenda in placebo studies needs to be expanded.

**Table 1:** Placebos and nursing: motivating empirical research.

**Educational and practice considerations**	Empirical research questions^*^
Knowledge	i. What is your understanding of the placebo and nocebo effects in clinical practice? ii. What is your understanding of the role of placebos in clinical practice? iii. What is your understanding of the role of placebos in research contexts?
Practices	iv. Have you ever made use of placebo effects in clinical practice? • If “yes,” in what ways did you attempt to foster placebo effects? v. Have you ever attempted to reduce the risk of nocebo effects in clinical practice? • If “yes,” in what ways did you attempt to mitigate nocebo effects? vi. Have you ever prescribed impure or pure placebos to patients? • If “yes,” how often have you prescribed impure/pure placebos to patients? • If “yes,” under what circumstances have you prescribed impure/pure placebos? • If “yes,” for what conditions/patients have you prescribed impure/pure placebos? vii. Have you witnessed physicians prescribe placebos? viii. Have you ever been asked to prescribe placebos by a physician?
Attitudes	ix. Do you believe it is acceptable to use placebos in clinical practice? • If “yes,” under what circumstances might placebos be acceptable? x. Do you believe placebo effects should be maximized in clinical care? xi. Do you believe that nocebo effects should be minimized? xii. Should training and education about placebo concepts be taught to nurses?

* *We advocate: (a) the use of both closed-question and qualitative surveys to ascertain the views of nurses; and (b) cross-cultural data collection*.

## Conclusions and Recommendations

Scientific understanding of placebo and nocebo effects has burgeoned in recent years. In accordance with empirical advancements, considerable ethical attention has been given to placebo and nocebo effects, and the use of placebos in clinical practice. However, this research has almost exclusively been refracted through the prism of doctors' practices. The vast majority of the studies conducted to date have predominantly focused on the knowledge and attitudes of doctors. It is unclear why this gap in nursing and placebo research has persisted (32). Yet non-physician health professionals are no less likely than doctors to face situations in which they can influence patients' health outcomes through placebo and nocebo effects or by the routine prescription of placebos. We strongly recommend that greater attention be paid to the attitudes and understanding of placebo concepts among nurses. Beyond determining their views and experiences, it will also be important to compare knowledge, and practices with those of physicians; e.g., the use of placebos and non-specific treatments varies among different areas of practice (48). It would also be important to investigate whether differences can be found across different nursing specialties. Future research might investigate the differences in scope and size of placebo effects, when delivered by nurses (or other personnel) rather than physicians. In turn, these research findings could inform nursing education on placebo/nocebo. Finally, the practice implications of placebo studies do not end with doctors and nurses: we emphasize that future research should also encompass deeper understanding of the role of physician assistants, clinical psychologists, psychotherapists, occupational therapists, and a host of other valuable members of care teams.

## Author Contributions

CB conceived the manuscript. MA, SB, and CB wrote the first draft. MA, SB, and CB revised the first draft. All authors approved the final version.

## Conflict of Interest

SB PhD candidate in psychology, MA healthcare ethicist, CB philosopher of medicine, SS-F MA in philosophy and anthropology.
